# Transcatether closure of an atrial redirection baffle leak

**DOI:** 10.4103/0974-2069.52819

**Published:** 2009

**Authors:** Brian D Soriano, Karen K Stout, Colleen D Cailes, Thomas K Jones

**Affiliations:** 1 Division of Cardiology, Department of Pediatrics, Seattle Children's Hospital, Washington, USA; 2 Division of Cardiology, Department of Medicine, University of Washington Medical Center, University of Washington School of Medicine, Seattle, Washington, USA

**Keywords:** Echocardiography, three-dimensional echocardiogarphy, transesophageal heart catheterization

## Abstract

We present the case of a woman born with transposition of the great arteries, who was surgically repaired with the Mustard technique. Because of interatrial shunting, she was brought to the cardiac catheterization laboratory for device closure. Matrix-array 3D transesophageal echocardiography enabled visualization of both baffle leaks and demonstrated its orientation in a fashion superior to 2D imaging, had it been used alone. The leaks were successfully closed with a single transcatheter device.

## INTRODUCTION

Before the current era of the arterial switch operation, “atrial redirection” procedure for patients born with transposition of the great arteries (d-TGA) was commonly performed beginning in 1958 through the early 1990s. Two different atrial redirection operations — the Senning and Mustard procedures — had been developed to channel blood flow into the appropriate ventricles, allowing correction of the intense cyanosis that characterizes this condition. The two operations differ in the way the baffle is constructed, but residual or recurrent baffle leaks can occur with both procedures. Such leaks account for roughly 60% of the late reoperations.[1]

## CASE REPORT

One such patient at our institution was a 32-year-old woman born with d-TGA and pulmonary stenosis, who underwent the Mustard procedure at 1 year of age. She had reestablished care after a 4-year absence and was reportedly asymptomatic, although exercise testing suggested moderately diminished exercise capacity. Transthoracic echocardiography detected a 1 cm interatrial baffle leak with pulmonary venous to systemic venous shunting (“left to right” or “oxygenated to deoxygenated”). The pulmonary valve was dysplastic, with both stenosis and severe regurgitation; the peak velocity across the pulmonary valve being 3.6 m/s. By cardiac magnetic resonance imaging, her measured Qp:Qs was 1.5:1.0. The left (pulmonary) ventricle systolic function was normal but dilated, with an end diastolic volume of 118 ml/m^2^.

**Figure 1:**
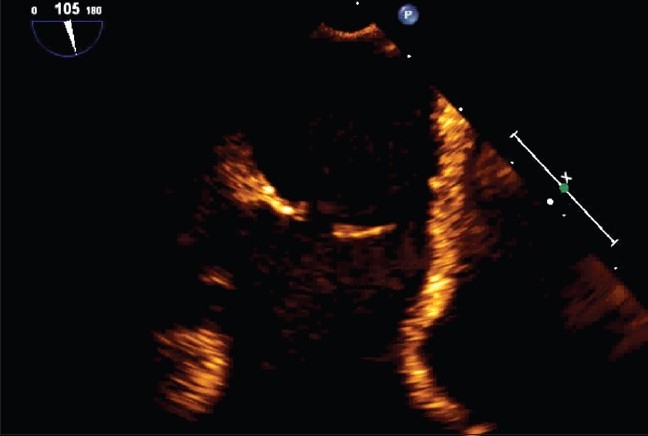
Two-dimensional image of the interatrial baffle and the two fenestrations

**Figure 2:**
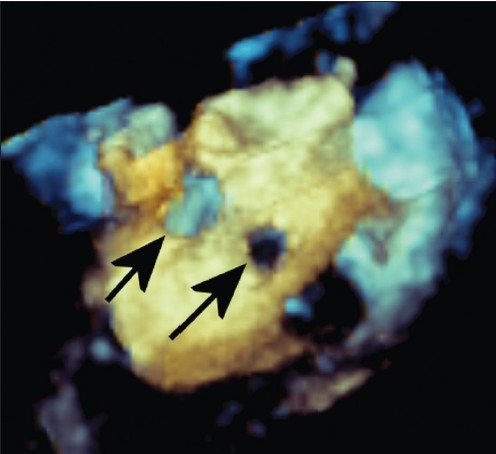
Matrix-array three-dimensional transesophageal echocardiogram obtained at the level of the mid esophagus, directly behind the anatomic left atrium. One larger and one smaller baffle leak are demonstrated (arrows)

**Figure 3:**
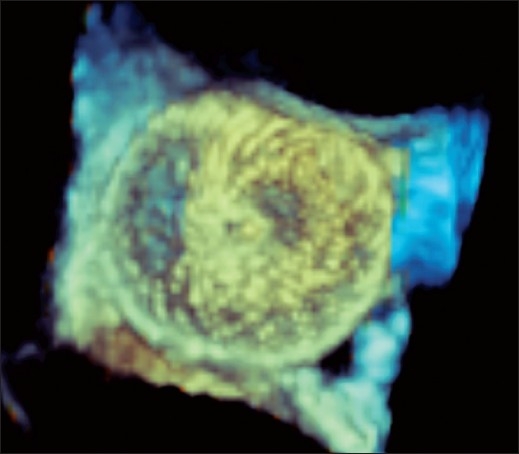
Still-frame image of the Amplatzer device after it has been deployed through the larger fenestration

Because of the concern of the volume-loaded left ventricle from her pulmonary insufficiency and her atrial level shunt, she was referred for cardiac catheterization and transcatheter closure using transesophageal echocardiography (TEE) assistance. After initiating general anesthesia, a TEE probe with a matrix-array transducer and real-time three-dimensional imaging capability (3D TEE) was placed. Two separate defects were detected in the superior limb of the atrial baffle. The superior defect measured 9 × 10 mm and a smaller, more inferior defect measured 4 × 4 mm. The distance between the two defects was 9 mm and the entire area was well demonstrated by both 2D [Figure 1] and 3D TEE [Figure 2; Movie 1 and 2]. Through the right internal jugular venous access, a 13 mm Amplatzer Septal Occluder (AGA Medical Corporation, Plymouth, MN) was deployed through the larger defect, completely closing it [Figure 3 and Movie 3]. The smaller defect was covered by a majority of the rim of the occluder, with near-total reduction in the degree of shunting through the smaller defect.

## DISCUSSION

During TEE-assisted interventions, whether in the cardiac catheterization laboratory or in the operating room, the echocardiographer performing the TEE must acquire images using 2D sweeps, mentally reconstruct the anatomy and then verbally report the findings to the surgeon or interventional cardiologist. As an alternative, real-time 3-dimensional echocardiography (3DE), using a matrix-array transducer, can quickly create a simulated 3D picture. In addition to describing the anatomy, the imaging cardiologist can efficiently display the 3D images to the interventionalist or surgeon, possibly introducing additional clinical and diagnostic benefits.

Other institutions have reported the successful use of 3D TEE during percutaneous interventions such as atrial septal defect closures[2,3] and electrophysiological procedures.[4] 3DE has been documented in other arenas such as perioperative assessment during pediatric aortic valve repair[5] and ventricular volume measurements.[6,7] In addition to 3D imaging, the matrix- array TEE transducer used in this report integrates more traditional echocardiography modalities such as 2D, M-mode and Doppler imaging.
